# A Case of Subclinical Hypothyroidism with Lingual and Right Pretracheal Ectopic Thyroid

**DOI:** 10.4274/jcrpe.1791

**Published:** 2015-06-03

**Authors:** Min Sun Kim, Young Hwa Kong, Dae-Yeol Lee

**Affiliations:** 1 Chonbuk National University Medicine School, and Research Institute of Clinical Medicine of Chonbuk National University-Biomedical Institute of Chonbuk National University Hospital, Department of Pediatrics, Jeonju, Korea

**Keywords:** hypothyroidism, mass, dual ectopic thyroid

## Abstract

Ectopic thyroid tissue is most commonly located in a single location, this being the lingual area. Presentation with two ectopic thyroid foci is quite unusual. A girl patient aged 7 years who presented with complaints of two masses in the right anterior neck and submandibular area is reported. Her growth pattern and development were normal. The masses were detected to be dual ectopic thyroid glands by ultrasonography, computed tomography and 99m-technetium pertechnetate thyroid scan. The patient also had subclinical hypothyroidism. She was treated with oral levothyroxine and the masses slightly decreased in size. The repeated thyroid function tests were within the normal limits. Thyroid function tests and imaging studies need to be conducted in all patients with anterior neck masses.

## INTRODUCTION

The thyroid gland is the first endocrine gland to appear during embryonic development. It begins to develop about 24 days after fertilization from a median endodermal thickening in the floor of the primitive hypopharynx; the endodermal thickening forms the thyroid diverticulum. Until the seventh embryonal week, the developing thyroid gland descends in the neck, passing ventrally towards the developing hyoid bone and laryngeal cartilages. Normally, the final location of the gland is in front of the developing trachea at the level of the second and third tracheal cartilages. The prevalence of ectopic thyroid gland is approximately 1 per 100 000 to 300 000 and it reportedly occurs in 1 of 4000 to 8000 patients who have thyroid disease (1). Here, we describe a case of dual ectopic thyroid in the lingual and right pretracheal regions presenting as a suddenly enlarged neck mass.

## CASE REPORT

**Figure 1:**
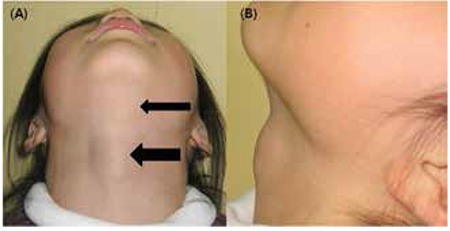
Inspective findings of patient showing 2 masses on the right upper neck and left submandibular area (2 arrows) (A. anterior view, B. lateral view).

A 7-year-old girl presented with a 1-week history of swelling in the right anterior neck and submandibular area. Her past history and family history were noncontributary. Her growth pattern and development were also normal. Inspection of the neck revealed swellings in the right upper and left submandibular areas with no distortion of the overlying skin (Figure 1). Physical examination revealed a fixed, hard, mildly tender pretracheal mass, 1.5x2.0 cm in size and another fixed, hard submandibular mass, 0.5x0.5 cm in size. There was no enlargement of the cervical lymph nodes. The remaining findings of the physical examination were unremarkable.

**Figure 2:**
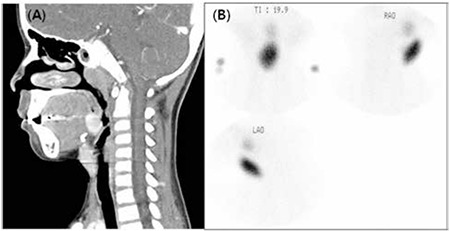
Neck computed tomography (A) and 99m-technetium pertechnetate thyroid scintigraphy (B) showing dual ectopic thyroid on lingual and right pretracheal portions.

The masses were evaluated using ultrasonography (USG) and computed tomography (CT). USG examination revealed well-defined masses with heterogeneous echogenicity and the thyroid gland was not in the expected cervical location. Neck CT revealed aberrant thyroid tissue as a 1.3x2.3 cm, round-shaped, high-density mass in the right pretracheal area and another 1.4x1.6 cm mass with the same characteristics in the region of the tongue base (Figure 2A). A 99m-technetium pertechnetate thyroid scintigraphy was performed, which demonstrated two ectopic areas of uptake (upper anterior neck and lingual areas), confirming dual thyroid ectopia (Figure 2B). There was no uptake in the normal anatomical location of the thyroid gland. Based on these findings, a diagnosis of dual ectopic thyroid was made. Biochemical determinations were consistent with a state of subclinical hypothyroidism. Serum triiodothyronine level was 1.7 ng/mL (normal, 0.9-2.4 ng/mL); free thyroxine (fT4), 17.2 pmol/L (normal, 10-28 pmol/L) and thyroid-stimulating hormone (TSH) level was 9.49 mIU/L (normal, 0.7-6.4 mIU/L).

Following the diagnosis, the patient was treated conservatively with oral levothyroxine, 0.05 mg per day. After treatment, she remained asymptomatic, the masses slightly decreased in size and repeated thyroid function tests were found to be within the normal limits.

## DISCUSSION

A normal thyroid gland migrates from the foramen cecum to its final pretracheal position. Ectopic thyroid tissue is the result of abnormal embryologic development/migration of the gland and can be observed anywhere along the descending pathway of the gland. The prevalence of ectopic thyroid tissue is 7-10% and dual ectopic thyroid is very rare (2).

An ectopic thyroid gland, first described by Hickman in 1869 (3), is defined as thyroid tissue not located anterolaterally at the level of the second to fourth tracheal cartilages. Ectopic thyroid glands typically become evident during adolescence or pregnancy when the requirement for thyroid hormones increases, leading to an elevation in the circulating TSH levels and an increase in the size of ectopic thyroid tissue (4). This course presents clinically as an enlarging neck mass. Although the majority of patients with ectopic thyroid tissue are usually asymptomatic, the present patient complained of a hard, fixed, palpable neck mass. Because subclinical hypothyroidism (elevated TSH and normal fT4 levels) was observed, the initial levothyroxine dose was set at the conventional total daily dose, which can be as low as 0.05 mg; this resulted in improvement in the mass lesion and thyroid function tests.

The most common ectopic location of the thyroid is in the lingual area, but it can also be the mediastinum, heart, esophagus and larynx (5). However, lesions located lateral to the midline of the trachea are very rare. Differential diagnosis based on the location of the neck mass can vary. Masses located at the base of the tongue include lingual ectopic thyroid and should be differentiated from mucus retention cysts, vallecular cysts and hypertrophic lingual tonsils. Lateral neck masses can be due to any of the following; cystic lymphangioma, lipoma, epidermoid cyst, vascular malformation, ectopic thyroid, enlarged lymph node or malignancy (6). Therefore, in addition to considering age of the patient and the characteristics of the mass, cervical USG, CT or magnetic resonance imaging is often required. Suspicion of malignancy may require fine-needle or incisional/excisional biopsy for confirmation. Before excisional biopsy, a thyroid scan should be performed in all cases to avoid permanent iatrogenic hypothyroidism.

In conclusion, although dual ectopic thyroid is rare, it does occur and a diagnosis of ectopic thyroid should be considered when evaluating neck masses. In the present case, subclinical hypothyroidism was also present along with a dual ectopic thyroid. We recommend that in addition to imaging studies, thyroid function tests should be performed in all patients with ectopic thyroid glands.
